# Impact of infection timing before vaccination on the effectiveness of COVID-19 mRNA vaccine in healthcare workers

**DOI:** 10.1128/spectrum.01288-25

**Published:** 2025-11-12

**Authors:** Mika Takatori, Atsushi Nemoto, Yosuke Hirotsu, Takahiro Mikawa, Rie Honda, Miho Ono, Aiju Endo, Kensuke Shibamori, Ryota Shiozawa, Makoto Maejima, Masahiro Shibusawa, Yume Natori, Yuki Nagakubo, Kazuhiro Hosaka, Minako Hoshiai, Yoshihiro Miyashita, Masakiyo Wakasugi, Hitoshi Mochizuki, Masao Omata

**Affiliations:** 1 Division of Infection Control and Prevention, Yamanashi Central Hospital 13643, Kofu, Yamanashi, Japan; 2 Department of Neonatology, Yamanashi Central Hospital 13643, Kofu, Yamanashi, Japan; 3 Genome Analysis Center, Yamanashi Central Hospital 13643, Kofu, Yamanashi, Japan; 4 Department of General Medicine and Infectious Diseases, Yamanashi Central Hospital 13643, Kofu, Yamanashi, Japan; 5 Nursing Bureau, Yamanashi Central Hospital 13643, Kofu, Yamanashi, Japan; 6 Central Clinical Laboratory, Yamanashi Central Hospital 13643, Kofu, Yamanashi, Japan; 7 Department of Pharmacy, Yamanashi Central Hospital 13643, Kofu, Yamanashi, Japan; 8 Medical Affairs Division, Yamanashi Central Hospital 13643, Kofu, Yamanashi, Japan; 9 Division of Microbiology in Clinical Laboratory, Yamanashi Central Hospital 13643, Kofu, Yamanashi, Japan; 10 Division of Genetics and Clinical Laboratory, Yamanashi Central Hospital 13643, Kofu, Yamanashi, Japan; 11 Department of Pediatrics, Yamanashi Central Hospital 13643, Kofu, Yamanashi, Japan; 12 Lung Cancer and Respiratory Disease Center, Yamanashi Central Hospital 13643, Kofu, Yamanashi, Japan; 13 Division of Nephrology, Department of Internal Medicine, Yamanashi Central Hospital 13643, Kofu, Yamanashi, Japan; 14 Department of Gastroenterology, Yamanashi Central Hospital 13643, Kofu, Yamanashi, Japan; 15 The University of Tokyo, Bunkyo-ku, Tokyo, Japan; Instituto Nacional de Tecnologia Agropecuaria, Buenos Aires, Argentina

**Keywords:** vaccine, SARS-CoV-2, healthcare worker, booster, infection history

## Abstract

The coronavirus disease 2019 (COVID-19) outbreak has prompted various public health interventions, including widespread vaccination campaigns. While vaccine effectiveness is widely studied, limited evidence exists on how it is influenced by the timing of previous severe acute respiratory syndrome coronavirus 2 (SARS-CoV-2) infection. This study aimed to evaluate the impact of prior infection timing on the effectiveness of subsequent COVID-19 mRNA vaccination in preventing SARS-CoV-2 infection. We conducted a cohort study of healthcare workers during the 10th wave of COVID-19 in Japan (January–July 2024) to evaluate whether prior infection history influences subsequent infection rates. Participants were categorized based on sex, age, vaccination status, and prior infection history, which was classified as infected within the past year (January to December 2023), infected more than a year ago (before December 2022), or never infected. Infection rates were compared among these groups. Vaccination in 2023 was associated with a significant reduction in infection rates across all groups, with a notable reduction in those infected before 2022 (risk ratio: 0.36, 95% CI: 0.19–0.66). The strongest protective effect was observed among individuals whose last infection occurred more than one year prior to vaccination. In contrast, vaccination showed a less substantial effect on individuals who had been infected within one year prior to receiving the vaccine. These findings suggest that COVID-19 vaccination remains a critical strategy for preventing infection, particularly among individuals with prior infection occurring more than 1 year earlier.

**IMPORTANCE:**

Understanding how prior infection timing affects the effectiveness of booster vaccination is essential for optimizing COVID-19 immunization strategies. While mRNA vaccines have demonstrated strong protection, their impact may vary depending on the interval between natural infection and vaccination. This study evaluates vaccine effectiveness among healthcare workers stratified by infection history, revealing that individuals whose last infection occurred more than one year before vaccination experienced the greatest benefit. In contrast, those with recent infections showed a less pronounced response. These findings suggest that immune memory and timing of antigen exposure play a critical role in booster performance. By identifying subgroups with differential vaccine responsiveness, this research provides actionable insights for tailoring booster recommendations, particularly during periods of high transmission risk.

## INTRODUCTION

The health and safety of healthcare workers (HCWs) constitute a critical priority in the management of the patients infected with severe acute respiratory syndrome coronavirus 2 (SARS-CoV-2) (1, 2). Protecting healthcare personnel is essential not only for mitigating their personal risk but also for ensuring the continuity of healthcare services. Disruptions caused by infections among HCWs can lead to workforce shortages and impair the delivery of essential medical care. Furthermore, infected HCWs may become a significant vector for intra-facility transmission, posing heightened risks to vulnerable patient populations, including cancer patients and those with immunodeficiency, as well as other staff members (3–7). Thus, evaluating the effectiveness of mRNA vaccines in high-risk HCWs is imperative to refine public health strategies and optimize vaccination policies.

The effectiveness of mRNA vaccines in reducing SARS-CoV-2 infection rates has been well-known in the general population and HCWs (8–12). Recent meta-analytic evidence indicates that vaccine effectiveness against SARS-CoV-2 infection declines substantially beyond 6–9 months after the last immunological event, emphasizing the importance of infection timing in evaluating booster response (13). However, the degree to which the timing of past infection affects vaccine-induced protection is not well understood, and it remains unclear which groups benefit most from booster vaccination.

To address the need for evidence-based vaccination strategies in healthcare settings, we conducted an observational study using a cohort of HCWs with a reactive in-hospital testing system triggered by symptom onset or known exposure with other COVID-19 patients and documented vaccination records. The primary objective of this study was to evaluate how the timing of prior SARS-CoV-2 infection influences the effectiveness of subsequent mRNA vaccination in preventing SARS-CoV-2 infection during the 10th wave in Japan. Insights from this study are expected to inform targeted booster recommendations and enhance protection strategies for HCWs during periods of elevated transmission risk.

## MATERIALS AND METHODS

### Study population and data collection

This study was conducted as a retrospective cohort study. This study included 1,211 HCWs employed at Yamanashi Central Hospital who remained continuously affiliated from 1 April 2023 to 1 August 2024. Participants included physicians, nurses, technicians, and administrative staff (Table 1). The 7-month observation period for assessing vaccine effectiveness spanned from 1 January 2024 to 31 July 2024. To prevent the spread of nosocomial infections, HCWs who tested positive for SARS-CoV-2 were required to promptly report their status to the hospital’s infection control nurse specialist, as part of the hospital’s infection control policy. SARS-CoV-2 testing has been conducted through a reactive in-hospital system since 2020, triggered by symptom onset or identification as a close contact. This diagnostic approach was consistently applied throughout the study period, including for determining infection history in 2022 and 2023.

**TABLE 1:** Baseline characteristics, occupations, and infection status

Characteristic	Number of healthcare workers (*n* = 1,211)
Gender, *n* (%)	
Male	342 (28.2%)
Female	869 (71.8%)
Age, median (range)	33 (19–78)
Occupation, *n* (%)	
Physicians	144 (11.9%)
Nurses	650 (53.7%)
Nursing assistants	66 (5.5%)
Pharmacists	41 (3.4%)
Clinical laboratory technicians	43 (3.6%)
Radiology technicians	31 (2.6%)
Clinical engineers	23 (1.9%)
Physical therapists	14 (1.2%)
Administrative staff	127 (10.5%)
Others	72 (5.9%)
Prior infection status, *n* (%)	
No infection history	608 (50.2%)
Last infection in 2022 or earlier	254 (21.0%)
Last infection in 2023	349 (28.8%)
Vaccination history prior to 2022, *n* (%)	
Vaccinated	1,128 (93.1%)
Not vaccinated	83 (6.9%)
Infection status during observation period, *n* (%)	
Infected	243 (20.1%)
Not infected	968 (79.9%)

HCWs were classified as “infected” if they were diagnosed through hospital-based testing, external medical institutions, or self-administered antigen test kits. Although most cases were symptomatic or close contacts with COVID-19 cases, a small number of asymptomatic infections were also included based on clinical judgment or workplace exposure concerns. Both symptomatic and asymptomatic infections were included in the analysis.

The infection control nurse directly interviewed infected HCWs via telephone to collect comprehensive data, including details about symptoms, presumed routes of transmission, vaccination history, and the date of symptom onset. Vaccination history, including dates and types of vaccines received, was obtained from the hospital’s occupational health vaccination records. Close contact was defined by criteria, such as (i) unmasked conversations or shared meals at close range, (ii) cohabitation or prolonged exposure in enclosed spaces, (iii) direct contact with respiratory secretions or bodily fluids. Participant age was recorded as of 1 January 2024.

Individuals were classified as having no history of infection if they had no documented positive SARS-CoV-2 test result. This group included those who were asymptomatic and never tested, as well as those who underwent testing due to symptoms or exposure but consistently tested negative. Routine testing of asymptomatic HCWs was not implemented during the study period. Therefore, it is possible that undetected infections occurred among individuals classified as having no history of infection.

### Regional infection data

Information on SARS-CoV-2 prevalence in Yamanashi prefecture was obtained from the Yamanashi Infectious Disease Control Center. Data were collected from sentinel surveillance conducted at 41 designated medical facilities, primarily clinics, and were aggregated by the prefectural government. From 1 July 2022 to 8 May 2023, infection data were comprehensively reported. Following the reclassification of COVID-19 as a category five infectious disease on 8 May 2023, fixed point surveillance reporting was implemented from 9 May 2023 to 31 July 2024.

### SARS-CoV-2 test

Several molecular diagnostic platforms for nucleic acid amplification and antigen testing were used to diagnose SARS-CoV-2 infection. The diagnostic tests used were TaqMan real-time reverse transcription-polymerase chain reaction (PCR) targeting the nucleocapsid gene in the StepOnePlus real-time PCR system (Thermo Fisher Scientific, Waltham, MA, USA) according to the protocol developed by the National Institute of Infectious Diseases in Japan (14), the FilmArray Respiratory Panel 2.1 test in the FilmArray Torch system (bioMérieux, Marcy-l’Etoile, France), the Xpert Xpress SARS-CoV-2 test in the Cepheid GeneXpert system (Cepheid, Sunnyvale, CA), cobas SARS-CoV-2 and influenza A/B in the cobas Liat system (Roche Diagnostics, Basel, Switzerland), and the Lumipulse antigen test in the LUMIPULSE G600II system (Fujirebio, Inc., Tokyo, Japan) (15, 16). Screening tests for close contacts and suspected cases were performed by pooling PCR (17). All tests were performed with material obtained from nasopharyngeal swabs immersed in viral transport medium, including UTM Viral Transport (Copan, Murrieta, CA) or ALLTM set medium (SG Medical, Seoul, Republic of Korea). These diagnostic tests represent the standard methods used for SARS-CoV-2 detection at our hospital and are commonly employed in clinical practice in Japan.

### Vaccination

HCWs were offered vaccinations on a voluntary basis through the hospital’s regular vaccination program. In May 2023, they received the bivalent mRNA vaccine targeting BA.4/BA.5 variants (Pfizer-BioNTech), and in November 2023, the monovalent mRNA vaccine targeting the XBB variant (Pfizer-BioNTech) was administered.

### Statistical analysis

Vaccination history, infection history, and related data were organized and aggregated using Microsoft Excel. To evaluate how the timing of prior SARS-CoV-2 infection influenced the effectiveness of mRNA vaccination, participants were stratified by infection history and vaccination status in 2023. Infection history was categorized into three groups: (i) “No infection history,” referring to individuals with no documented infection between 2020 and 2023; (ii) “Last infection in 2022 or earlier,” referring to individuals with at least one documented infection between 2020 and the end of 2022 but none in 2023; and (iii) “Last infection in 2023,” referring to individuals with at least one documented infection between January and December 2023, regardless of any earlier infection history. Infection rates were compared across these subgroups. Univariate analyses were conducted using the χ test. Multivariable logistic regression was performed to assess the independent effects of infection timing and vaccination status, adjusting for sex, age group, and prior vaccination before 2022. Statistical analyses were performed with IBM SPSS Statistics (version 27). Statistical significance was defined as a *P*-value < 0.05.

## RESULTS

### Study cohort

This study included a cohort of 1,211 HCWs affiliated with our hospital (Table 1). The cohort comprised 342 males (28.2%) and 869 females (71.8%), with a median age of 33 years (range: 19–78). The distribution of roles among the participants was as follows: 144 physicians, 650 nurses, 66 nursing assistants, 41 pharmacists, 43 clinical laboratory technicians, 31 radiologic technologists, 23 clinical engineers, 14 physical therapists, 127 administrative staff, and 72 individuals categorized as other. Among the cohort, 608 (50.2%) reported no history of infection, 254 participants (21.0%) had their last infection recorded before 31 December 2022, and 349 (28.8%) experienced infections during 2023 (between 1 January 2023 and 31 December 2023). In terms of vaccination history, 1,128 individuals (93.1%) had received at least one dose of a SARS-CoV-2 vaccine prior to 2023, while only 83 individuals (6.9%) had no documented vaccination before that year. During the observation period, 243 individuals (20.1%) were confirmed to have been infected with SARS-CoV-2, while 968 (79.9%) were not infected.

### Trends in infections and close contacts

**Fig 1:**
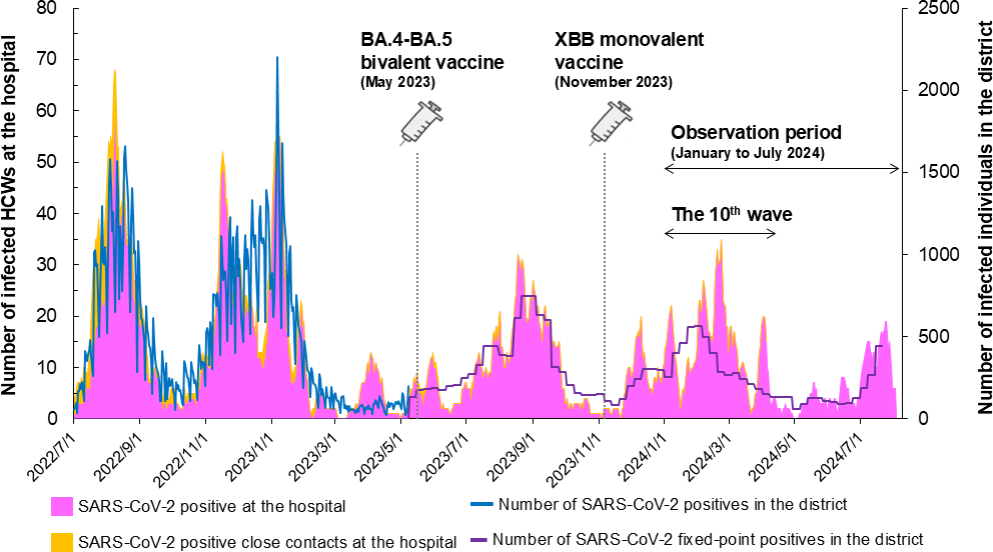
The changes in the number of SARS-CoV-2 infected individuals. The graph shows the number of healthcare workers (HCWs) at the hospital (left y-axis), categorized into SARS-CoV-2 positive individuals (magenta), and SARS-CoV-2 positive close contacts (orange). The blue and purple lines represent the number of SARS-CoV-2 positive cases in the district, and the number of fixed-point positive cases in the district, respectively (right y-axis). The bidirectional arrows show the duration of the 10th wave of infection and the observation period.

During the observation period, a significant surge in COVID-19 infections was observed following vaccination, coinciding with the widespread transmission during the 10th wave (approximately December 2023 to April 2024) (Fig. 1). Hospital staff who opted for vaccination in 2023 received either one or two doses of the mRNA vaccine, administered in May and November, respectively. Throughout this period, a total of 248 staff members tested positive for SARS-CoV-2, while 119 individuals were identified as close contacts.

### Preventive effect of vaccination against SARS-CoV-2 infection

To assess the preventive effect of vaccination against SARS-CoV-2 infection, we analyzed the relationship between prior infection history, vaccination status in 2023, and subsequent infection incidence during the observation period. Among unvaccinated individuals after 2023 (*n* = 623), infection rates were 29.7% (84/283) of those with no prior infection, 24.0% (35/146) of those infected in 2022 or earlier, and 12.9% (25/194) of those infected in 2023 (Table 2). In contrast, among individuals who received at least one vaccination after 2023 (*n* = 588), the infection rates were 23.1% (75/325) for those with no prior infection history, 9.3% (10/108) for those infected before 2022, and 9.0% (14/155) for those infected during 2023 (*P* < 0.05, χ² test) (Table 2). These findings suggest that vaccination slightly reduced infection rates among individuals with no prior history of SARS-CoV-2 infection and those infected during 2023 (i.e., within 1 year prior to the observation period starting January 2024). However, vaccination led to a more remarkable reduction in infection rates among individuals whose previous infection occurred before 2022 (i.e., more than one year prior to the start of the observation period), indicating a strong booster effect of the vaccine in this group.

**TABLE 2:** SARS-CoV-2 infection rate of healthcare workers during the 10th wave in Japan^*c*^

Prior infection status	Infection rate in HCWs who were unvaccinated in 2023 (*n* = 623)	Infection rate in HCWs who were vaccinated in 2023 (*n* = 588)
No infection history	29.7% (84/283)	23.1% (75/325)
Last infection in 2022 or earlier^*a*^	24.0% (35/146)	9.3% (10/108)
Last infection in 2023^*b*^	12.9% (25/194)	9.0% (14/155)

^*a*^ Defined as last recorded infection date on or before 31 December 2022.

^*b*^ Defined as last recorded infection date between 1 January 2023 and 31 December 2023.

^*c*^ HCWs, healthcare workers.

### Subgroup analysis and risk ratio

**Fig 2:**
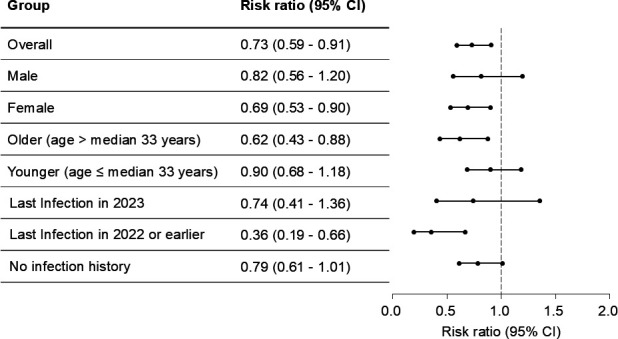
COVID-19 vaccine effectiveness stratified by gender, age, and infection history. The forest plot shows the risk ratio with 95% confidence intervals (CI) across different groups: overall population, males, females, individuals older than the median age (33 years), individuals younger than or equal to the median age, individuals infected in 2023, those infected in 2022 or earlier, and individuals with no prior SARS-CoV-2 infection. The dashed vertical line at a risk ratio of 1.0 represents no effect. The risk ratio and its 95% CI for each group are indicated.

To identify the factors contributing to the reduction in SARS-CoV-2 infection rates, we performed a subgroup analysis. In the overall cohort, recent vaccination significantly reduced the infection rate (risk ratio: 0.730, 95% CI: 0.587–0.909) (Fig. 2). Among subgroups, the reduction in infection rate was most pronounced in specific clinical backgrounds: females (0.691, 95% CI: 0.529–0.902), those older than the median age (0.616, 95% CI: 0.432–0.877), and those with a history of COVID-19 infection before 2022 (0.356, 95% CI: 0.191–0.664). Thus, it was found that the effect of vaccination was highest in individuals who had been infected more than 1 year previously, indicating the significant benefit of vaccination in this group.

### Multivariable analysis of infection risk

To assess the independent effects of prior infection on SARS-CoV-2 infection risk during the study period, we performed a multivariable logistic regression analysis. The model included infection history, vaccination status in 2023, sex, age group, and vaccination history prior to 2022. Both prior infection and vaccination in 2023 were significantly associated with reduced infection risk (adjusted OR = 0.577, 95% CI: 0.480–0.694, *P* < 0.001; and adjusted OR = 0.633, 95% CI: 0.469–0.854, *P* = 0.003, respectively) (Table 3). In contrast, sex, age group, and vaccination before 2022 were not significantly associated with infection risk. These findings indicate that both prior SARS-CoV-2 infection and mRNA vaccination in 2023 were independently associated with a reduced risk of infection during the study period.

**TABLE 3:** Multivariable logistic regression analysis of SARS-CoV-2 infection risk^*a*^

Variable	Adjusted OR	95% CI	*P*-value
Prior infection (2023 vs none)	0.577	0.480–0.694	<0.001
Vaccinated in 2023 (yes vs no)	0.633	0.469–0.854	0.003
Sex (male vs female)	1.001	0.728–1.377	0.994
Age (older vs younger)	0.84	0.624–1.130	0.249
Vaccinated before 2022 (yes vs no)	1.295	0.717–2.337	0.392

^*a*^ Reference categories: no prior infection, unvaccinated in 2023, female, younger age group, and no vaccination before 2022. OR, odds ratio; CI, confidence interval.

## DISCUSSION

This study examined the protective effect of mRNA vaccines against SARS-CoV-2 infection in HCWs, with a focus on the interplay between vaccination status and prior infection history. The findings demonstrate that vaccination significantly reduces the risk of reinfection, particularly among individuals whose prior infection occurred over a year ago. These findings suggest the sustained effectiveness of the mRNA vaccine in mitigating reinfection risk in HCWs.

The analysis demonstrated that the effectiveness of mRNA vaccination in reducing SARS-CoV-2 infection rates varied depending on the timing of prior infection, with greater protection observed in those infected more than a year before vaccination. Although this study did not examine the immunological status in HCWs, the observed reduction in infection risk may be associated with vaccine booster effects. The previous study indicated vaccination provided a strong booster effect for individuals with diminished immunity (18). Specifically, individuals who had been infected over a year ago experienced a marked reduction in infection rates following vaccination (risk ratio: 0.36, 95% CI: 0.19–0.66). Interestingly, HCWs who were vaccinated less than a year after infection showed a limited effect on reducing infection rates (0.74, 95% CI: 0.41–1.36) (Fig. 2). These findings indicate the importance of considering infection history when deciding the timing of booster vaccinations to maximize their effectiveness, especially in high-risk occupational environments. Previous studies have demonstrated that booster doses administered every 6 months to 1 year effectively reduce severe disease, particularly in older or immunocompromised populations (19). While breakthrough infections are known to occur, booster vaccinations remain critical for protecting against symptomatic disease (20–22). Namely, our findings emphasize the importance of considering infection history when developing vaccination strategies. The observed booster effect in individuals with infections more than one year prior indicates the importance of timely vaccination in maintaining immunity. However, as most participants in this study were under 65 years old, the duration of vaccine protection may be shorter in older populations. These insights underscore the need to tailor vaccination schedules to demographic and clinical factors to optimize protection, especially for high-risk groups.

This study has important implications for public health policy and future research. First, our findings reinforce the need for ongoing vaccination efforts, particularly in high-risk populations such as HCWs, who remain at the forefront of pandemic response and are frequently exposed to SARS-CoV-2. Second, the booster effect observed in individuals with a prior infection underscores the value of vaccination not only in preventing initial infection but also in providing durable protection against reinfection, especially for individuals who were infected more than 1 year ago. Future studies should explore the long-term effectiveness of vaccines in preventing reinfection, particularly in the context of emerging variants, and assess how prior infection history may influence vaccine effectiveness.

Several limitations warrant consideration. First, this study was conducted within a single healthcare institution, potentially limiting generalizability. Second, the relatively small sample size, particularly within specific subgroups, may limit the statistical power to detect smaller differences in effectiveness and the precision of our estimates. Third, self-reported vaccination and infection histories may introduce recall bias, despite rigorous data collection protocols. Furthermore, we were unable to stratify effectiveness estimates by the specific vaccine type (bivalent vs monovalent). Fourth, the lack of a quantitative assessment of spike antibodies in the blood precludes an evaluation of neutralizing activity against emerging SARS-CoV-2 variants. Finally, the observational design precludes definitive causal inference.

In conclusion, this study highlights the continued importance of COVID-19 vaccination in healthcare settings, even as the pandemic evolves. By demonstrating the significant benefit of vaccination, especially for those previously infected more than a year ago, we contribute to the growing evidence supporting vaccination as a key tool for preventing SARS-CoV-2 infection. These findings should inform future vaccination strategies, emphasizing the need for tailored approaches based on prior infection history to maximize the protective effect of vaccines.

## Data Availability

The data that support the findings of this study are not publicly available due to patient confidentiality but are available from the corresponding author upon reasonable request.
